# *In Vitro* and *In Vivo* Antitumor Activity of a Novel pH-Activated Polymeric Drug Delivery System for Doxorubicin

**DOI:** 10.1371/journal.pone.0044116

**Published:** 2012-09-24

**Authors:** Menglei Huan, Bangle Zhang, Zenghui Teng, Han Cui, Jieping Wang, Xinyou Liu, Hui Xia, Siyuan Zhou, Qibing Mei

**Affiliations:** 1 Department of Pharmaceutics, School of Pharmacy, Fourth Military Medical University, Xi'an, People's Republic of China; 2 Department of Pharmacy, Tangdu Hospital, Fourth Military Medical University, Xi'an, People's Republic of China; 3 Department of Thoracic-Cardio Surgery, First Affiliated Hospital of PLA General Hospital, Beijing, People's Republic of China; 4 Key Laboratory of Gastrointestinal Pharmacology of Chinese Materia Medica of the State Administration of Traditional Chinese Medicine, School of Pharmacy, Fourth Military Medical University, Xi'an, People's Republic of China; City of Hope Medical Center and Beckman Research Institute, United States of America

## Abstract

**Background:**

Conventional chemotherapy agent such as doxorubicin (DOX) is of limited clinical use because of its inherently low selectivity, which can lead to systemic toxicity in normal healthy tissue.

**Methods:**

A pH stimuli-sensitive conjugate based on polyethylene glycol (PEG) with covalently attachment doxorubicin via hydrazone bond (PEG-hyd-DOX) was prepared for tumor targeting delivery system. While PEG-DOX conjugates via amid bond (PEG-ami-DOX) was synthesized as control.

**Results:**

The synthetic conjugates were confirmed by proton nuclear magnetic resonance (NMR) spectroscopy, the release profile of DOX from PEG-hyd-DOX was acid-liable for the hydrazone linkage between DOX and PEG, led to different intracellular uptake route; intracellular accumulation of PEG-hyd-DOX was higher than PEG-ami-DOX due to its pH-triggered profile, and thereby more cytotoxicity against MCF-7, MDA-MB-231 (breast cancer models) and HepG2 (hepatocellular carcinoma model) cell lines. Following the *in vitro* results, we xenografted MDA-MB-231 cell onto SCID mice, PEG-hyd-DOX showed stronger antitumor efficacy than free DOX and was tumor-targeting.

**Conclusions:**

Results from these *in vivo* experiments were consistent with our *in vitro* results; suggested this pH-triggered PEG-hyd-DOX conjugate could target DOX to tumor tissues and release free drugs by acidic tumor environment, which would be potent in antitumor drug delivery.

## Introduction

Conventional chemotherapy agent such as doxorubicin (DOX) is of limited clinical use because of its inherently low selectivity, which can lead to systemic toxicity in normal healthy tissue. This prevents the use of giving high but effective doses for cancer treatment [1]–[3]. To overcome this problem, several high molecule drug carriers have been designed to deliver antitumor agents passively by targeting tumor tissue or cells using the enhanced permeability and retention effect (EPR effect) with favorable biocompatibility and solubility properties [4]. PEG has been widely used as a drug carrier in many drug design strategies for its low immunogenicity and long circulation time, antitumor agents conjugated to PEG are of great interest because of the passive tumor targeting by EPR effect and slow-released profile, led to a low toxicity, high efficacy and long-acting delivery vehicle [5]–[6].

Recently, stimuli-sensitive carriers have emerged and attracted great attention for modulating anticancer drug release by appropriate stimulus (temperature, pH etc), which can led to obvious enhanced therapeutic efficacy and low side-effects [7]–[10]. Considered the mild acidic extracellular microenvironment in tumor tissues and acidic lysosomes or endosomes in cells, pH-sensitive carriers have been ideal for selective release of antitumor agents in tumor tissues and/or within tumor cells. Many strategies have been applied in pH-triggered delivery system [11]. Among the strategies, hydrazone bond (R_1_R_2_C = NR) has been chosen for its pH-controlled hydrolysis. Polymers linked with hydrazone bond keep stable in physiological condition, once the pH value decrease to 5.0–6.0, the acid-sensitive bond between carrier and drug become unstable and then release free drug quickly [12]. Based on this profile, conjugates via hydrazone can bring to a burst of anticancer drug release in acidic organelles after endocytosis by tumor cells, therefore provide a high drug concentration at cellular level to fulfill better therapeutic efficacy. As reported, even extremely multidrug resistant (MDR) cells can be killed at high drug concentration, so this strategy is also meaningful in MDR cells [13]–[15].

In this paper, we have designed and covalently synthesized a pH-sensitive PEG-hyd-DOX conjugate for stimuli release of DOX, characterized by NMR and HPLC. The pH-sensitive hydrolysis of PEG-hyd-DOX was confirmed *in vitro* by HPLC, the higher intracellular accumulation of DOX by PEG-hyd-DOX in tumor cells was determined by HPLC/MS/MS,its probably intracellular route was firstly distributed into acidic organelles and then release free drugs to their targeting site. The antitumor activity *in vitro* and *in vivo* was evaluated to access the efficacy using PEG acidic-sensitive conjugate as tumor-targeting delivery system. Evidence is provided that the conjugate could target tumor tissues, inhibit tumor growth, prolong the life of tumor-bearing mice and reduce cytotoxicity in normal tissues, as compared with using free DOX alone *in vivo*.

## Results

### Synthesis and characterization of DOX conjugates

**Figure 1:**
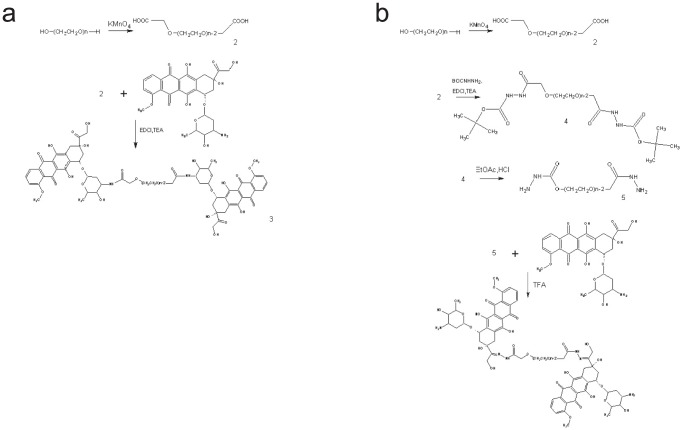
Synthetic schemes of (a) PEG-hyd-DOX and (b) PEG-ami-DOX.

The polymeric conjugates were synthesized as described above, shown in Fig. 1. For conjugation of DOX to PEG through hydrazone or amid bond, poly ethylene glycol was firstly oxide to poly ethylene glycol dioic acid catalyzed by KMnO_4_ at the terminal hydroxyl group. Then the hydrazone and amid conjugates were synthesized with help of catalyst, with yield of 82.5% and 87% respectively. The conjugates were purified by Sephadex G50 column eluted with water. The NMR spectrum (Fig. S1A) of PEG-hyd-DOX exhibited typical signals at chemical shifts of 7.1 ppm (2H, d, CH-2 and CH-4), 6.8 ppm (1H, d, CH-3), 5.3 ppm (1H, s, CH-1') and 4.08 ppm (3H, s, H_3_C-O-C-1), resulting from DOX, and at 3.61 ppm (PEG backbone); while the amid one exhibited typical signals at chemical shifts of δ7.8 (2H, d, CH-2 and CH-4), 7.2 (1H, d, CH-3), 5.40 (1H, s, CH-1'), 4.10 (3H, s, H3C-O-C-1), δ3.61 (PEG backbone). The DOX content of the hydrazone and amid conjugates were 10.64% and 3.9% (w/w) respectively, while the ideal conversion of DOX was about 15.34% and 16.16% for hydrazone and amid conjugates calculated by the molecule weight using the UV spectroscopic method (Figure S2), with absorbance of DOX at 480 nm. The purity of the two conjugates was all above 99% analyzed by HPLC, and typical chromatogram (Figure S1B) showed single sharp peak at 11.50 min (PEG-hyd-DOX) or 10.4 min (PEG-ami-DOX).

### *In vitro* drug release

**Figure 2:**
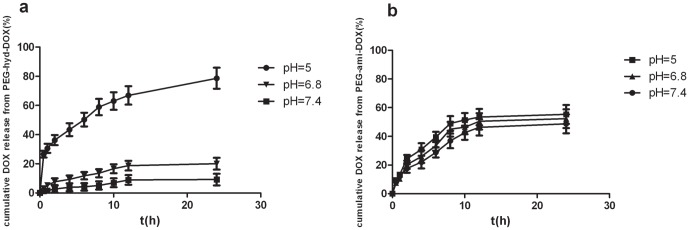
*In vitro* cumulative DOX release. (a) PEG-hyd-DOX and (b) PEG-ami-DOX at 37°C at different pHs (pH was 5.0, 6.8 and 7.4 respectively) which were analyzed by HPLC. (n = 4, mean ± SD).

The release behavior of the two conjugates was carried out in different pHs, acetate buffer (pH 5.0) and phosphate buffer (pH 6.8 or 7.4) at 37°C. Fig. 2a revealed a significantly pH-dependent release profile of PEG-hyd-DOX, when at pH 7.4, a physiological condition, the polymeric conjugates were seemed stable, after 24 h incubation, only about 10% free DOX released. However, when at pH 6.8, an extracellular tumoral condition [18], although mild acidic micro-environment occurred, still only about 20% cumulative DOX was determined after incubation. As turned to a modest acidic condition (pH 5.0), which was similar to intracellular acidic organelles pH value [19], the drug release became much fast with almost 80% cumulative DOX release after 24 h, presumably because of the pH-sensitive profile of the hydrazone linkage between DOX and polymer. On contrary, PEG-ami-DOX didn't show the same pH-liable release behavior. Fig. 2b showed PEG-ami-DOX were insensitive to the pH value, the DOX release rate was nearly same at different pHs, after 24 h incubation, the cumulative release of DOX was only 50%, which were much lower than hydrazone conjugates released at pH 5.0, indicated amid ones might not release free DOX completely in tumor cells, led to a lower antitumor efficacy than hydrazone conjugates.

### *In vitro* cellular sub-distribution

**Figure 3:**
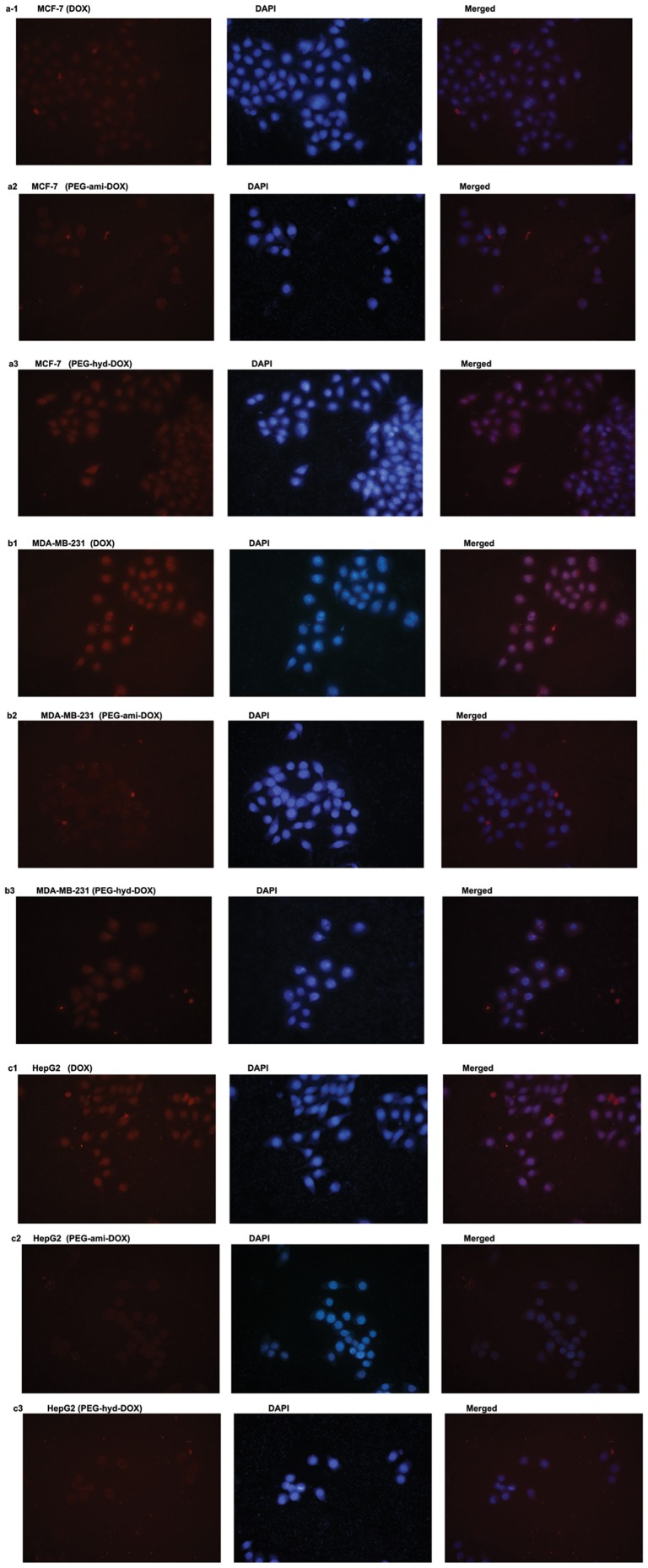
Fluorescence microscopy images of MCF-7, MDA-MB-231 and HepG2 Cells. Different cells were treated with 20 µM of DOX (a1,b1,c1), PEG-ami-DOX (a2,b2,c2), and PEG-hyd-DOX (c1,c2,c3) for 30 min, repectively. From left to right, there were images of DOX or its conjugates (red dots), DAPI staining for nucleus (blue dots) and their merged images.

In this study, the distribution of DOX and its conjugates in tumor cells were investigated using fluorescence microscopy by labeling with nucleus selective dye (DAPI, blue) and intrinsic red fluorescence of DOX. All three tumor cells exposed to free DOX showed an obvious fluorescence signal in the nucleus (evidenced by purple dots in nucleus, a sign of co-localization of DOX with DAPI) and also non-specificity sub-cellular distribution in cytoplasm after 30 min, which might be explained that the mechanism of drug action for DOX is mainly by interaction with topoisomerase II existed in nucleus and its cellular uptake mechanism was basically via diffuse. However, red fluorescent dots of its amid-linkage polymeric conjugates were almost observed in the cytoplasm (red), indicated they might be locked in cytoplasm after uptake, so an effective dose couldn't achieve at nucleus. Interestingly, the hydrazone linkage conjugates showed a much broad distribution either in cytoplasm (red) and nucleus (purple), confirmed the hydrolysis of hydrazone bond in tumor cells could release free drugs and help free DOX to distribute to its targeted sub-cellular region (nucleus) due to its pH-responsive profile, indicated the pH-triggered polymer via hydrazone bond could deliver drugs into tumor cell and then release the therapeutic agents to their targeting region as our designed (Fig. 3). And this phenomenon was in agreement with the results in the part of drug release experiment, such characteristic of drug distribution will be also tested and evidenced in the following experiments.

### LC/MS/MS analysis for intracellular DOX accumulation

The intracellular DOX levels in different tumor cell lines were analyzed by LC/MS/MS. Briefly, quantitation of DOX was performed by multiple reactions monitoring of the deprotonated precursor ion and the related product ion, using the internal standard method with peak area ratio. Collision-induced dissociation was achieved using argon as the collision gas. A standard solution of 1 µg/ml DOX and resveratrol (internal standard) was applied to optimize the detection condition in the presence of the mobile phase (acetonitrile: water = 70∶30, V/V). The compounds were separated on the C_18_ column using an isocratic mobile phase. The mass transitions used for DOX and resveratrol were *m/z* 542→395 (cone voltage, 120 eV; collision energy, 15 eV; dwell time, 400 ms) and 227→143 (cone voltage, 40 eV; collision energy, 30 eV; dwell time, 400 ms), respectively.

**Figure 4:**
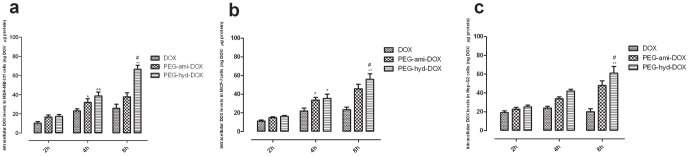
Intracellular DOX levels assayed by LC/MS/MS. After treatment by PEG-DOX conjugates (20 µM DOX. Eq) or DOX in MDA-MB-231 (left), MCF-7 (middle) or HepG2 cells (right) for 2, 4 and 8 h respectively. (^*^ *p*<0.05, ^**^ *p*<0.01, PEG-hyd-DOX *vs* DOX; ^#^ *p*<0.05, PEG-hyd-DOX *vs* PEG-ami-DOX, n = 4, mean ± SD).

**Figure 5:**
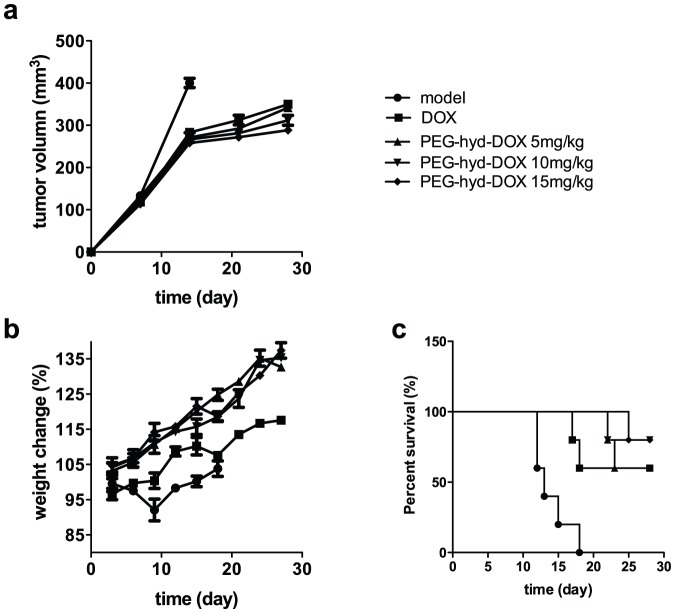
Evaluation of *in vivo* anti-tumor activity of free DOX and PEG-hyd-DOX (5,10,15 mg/kg DOX. eq) on nude mice after s.c. human breast MDA-MB-231 cells. (a) tumor volume curve; (b) body weight changes; (c) survival curve. (n = 5, mean ± SD).

After drug treatment with three tumor cells as described above for 2, 4, 8 h respectively, the intracellular DOX levels were analyzed by HPLC-MS-MS (Fig. 4). The intracellular DOX levels of two polymeric conjugates in the three tumor cells were both time-dependent increased, while free DOX didn't show this profile, which might be explained by the long circulation and slow-released profile of polymers. Both two polymeric DOX conjugates could increase the intracellular accumulation of DOX, and PEG-hyd-DOX led to a higher intracellular content of DOX due to the acid-liable hydrazone bond after taken up by cells. Therefore, the pH-triggered conjugates were expected to take on enhanced tumor cell accumulation and result in improved antitumor activity *in vivo* experiments. Among those three tumor cells, MDA-MB-231 was given the highest response to the polymeric drugs, which might be most sensitive to the synthesized conjugates *in vitro*. As shown in Fig. 4a, the intracellular DOX levels of two conjugates in MDA-MB-231 were not significantly higher than DOX at first 2 h, consistent with their slow-released mechanism [20]. As incubation time goes by, the difference of intracellular DOX levels between polymeric conjugates and free DOX became obvious (p<0.05 or 0.01), indicated an effective toxicity and therapeutic efficacy of our polymer needing much more treatment time. After 8 h incubation, the hydrazone treated group was 2.5 times higher than free DOX (p<0.01) and 1.8 times higher than amid conjugate (p<0.05), meant an obvious high accumulation of free DOX intracellular, and a mighty good biological activity of anticancer. Intracellular drug amount in MCF-7 cell was shown in Fig. 5b; it took on a similar trend as in MDA-MB-231cell, after 2 h treatment, there was no statistic improvement of cellular uptake by two DOX-conjugates; when treated after 4 h, both two polymer groups accumulated higher amount of DOX in cell than free DOX (p<0.05), however, the two conjugates made almost same performance (no statistical difference). Only after 8 h incubation, PEG-hyd-DOX could give a final significant difference compared with amid conjugate (p<0.05). When treated in HepG2 cell (Fig. 4c), the conjugates also showed a time dependent manner, and PEG-hyd-DOX showed the best response in drug cumulative among those three drugs, which could be 2.4 and 3 times higher than PEG-ami-DOX and DOX after 8 h incubation (p<0.05 or 0.01), similar with treatment on MDA-MB-231 and MCF-7 cells as described above, the two polymeric conjugates also didn't show an prominent performance at the beginning of the incubation time (2 h), their slow-released profile still was the explanation.

### *In vitro* cytotoxicity

From the above three different experiments, we found a common action on such drug release and distribution in tumor cell line, according to our original designing that hydrazone conjugates released much more free DOX than amid conjugates in the tumoral condition, and we will test the following hypothesis if such divergence will result in the different antitumor efficacy in *vitro*. The cytotoxic activity of PEG-hyd-DOX against MCF-7, MDA-MB-231 and HepG2 cells was determined using an MTT assay which was summarized in Table 1. PEG-hyd-DOX exhibited more toxicity than free DOX in growth inhibition of three tumor cells, while MDA-MB-231 cell was most sensitive, consistent to the results of LC/MS/MS analysis. The IC_50_ value of DOX against MCF-7, MDA-MB-231 and HepG2 cells were 1.15, 2.3, 1.9 times higher than PEG-hyd-DOX respectively. Although the intracellular drug accumulation of PEG-ami-DOX was higher than free DOX, but cytotoxicity of PEG-ami-DOX was lower than both DOX and PEG-hyd-DOX. In addition, the IC_50_ values of PEG-ami-DOX was 2.1, 4.3 and 2.6 times higher than PEG-hyd-DOX in MCF-7, MDA-MD-231 and HepG2 cells, respectively. The obvious difference in cytotoxicity among those three formulations indicated that high intracellular concentration may not always bring good performance in biological activity, an effective drug accumulation in site of action (with regard to DOX, nucleus is action region) must be most important.

**Table 1:** *In vitro* cytotoxicity of DOX and DOX-conjugates.

Drugs	IC_50_ (µM)		
	MCF-7	MDA-MB-231	HepG-2
DOX	8.3±0.2	10.2±0.3	14.3±0.4
PEG-hyd-DOX	7.2±1.1	4.4±0.6*	7.6±0.9*
PEG-ami-DOX	15.2±1.5	18.8±0.7	19.6±1.6

* *Significant effects of factors on student's t-test, p<0.05 compared with DOX. (n = 6, mean ± SD)*.

### *In vivo* antitumor activity

Based on our *in vitro* results and the clinical use of DOX, the *in vivo* experiments were evaluated by subcutaneously xenografting SCID mice with the human breast carcinoma cell line MDA-MB-231 cell. PEG-hyd DOX showed improved antitumor efficacy, lower toxicity, as measured in term of life span, tumor growth inhibition and body weight change, compared with free DOX.

Even given the same dose (5 mg/kg DOX equivalent), the tumor growth inhibition effect of PEG-hyd-DOX was better than that of free DOX at days 14, 21 and 28 after first drug treatment, as judged by the tumor volume (Fig. 5a). The antitumor activity took on a dose-dependent manner and showed a significantly difference between PEG-hyd-DOX (10, 15 mg/kg DOX equivalent) and DOX (5 mg/kg) 28 days later after therapy (p<0.05 or 0.01). Early in the treatment (days 7), the antitumor efficacy of PEG-hyd-DOX was not as good as DOX. This might be caused by the slow-released profile and intracellular uptake mode of polymer conjugate compared with that of free DOX, which was consistent with *in vitro* results.

Body weight change is a useful indicator of the systemic toxicity of the conjugates reference [21]. The body weight change curve showed that animals treated with PEG-hyd-DOX at three different doses gained weight steadily, however the body weight change was independent of dose. Conversely, mice treated with free DOX showed an initial decrease in body weight until day 6 and then a gradual weight gain; however they did not achieve similar level of weight gain as compared with the DOX conjugates treatment groups (Fig. 5b). These results showed that PEG-hyd-DOX could make effect without weight loss, which is a common side-effect in chemotherapy, suggested that a higher dose of DOX conjugate could potentially be used to give greater therapeutic efficacy, without serious side effects.

5, 10 and 15 mg/kg DOX conjugates groups (DOX equivalent) all could prolong the medium survival time to 26, 27 and 28 days respectively, as compared with mice treated with saline (13 days) or 5 mg/kg free DOX (24 days) (Fig. 5c).

### Pharmacokinetic and bio-distribution of PEG-hyd-DOX

**Figure 6:**
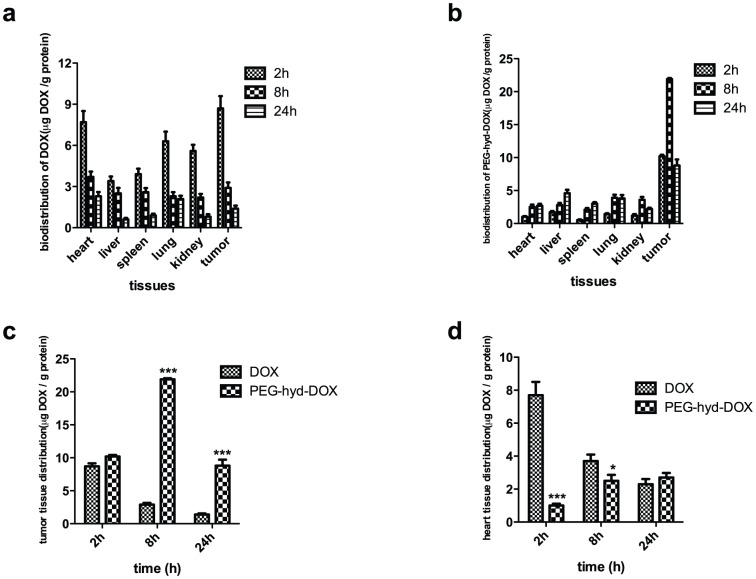
Bio-distribution and accumulation of free DOX and PEG-hyd-DOX (5,10,15 mg/kg DOX. eq) in tissues. (a) free DOX, (b)PEG-hyd-DOX; cumulative accumulation of DOX in (c) tumor and (d) heart assayed by LC/MS/MS. (n = 5, mean ± SD).

**Figure 7:**
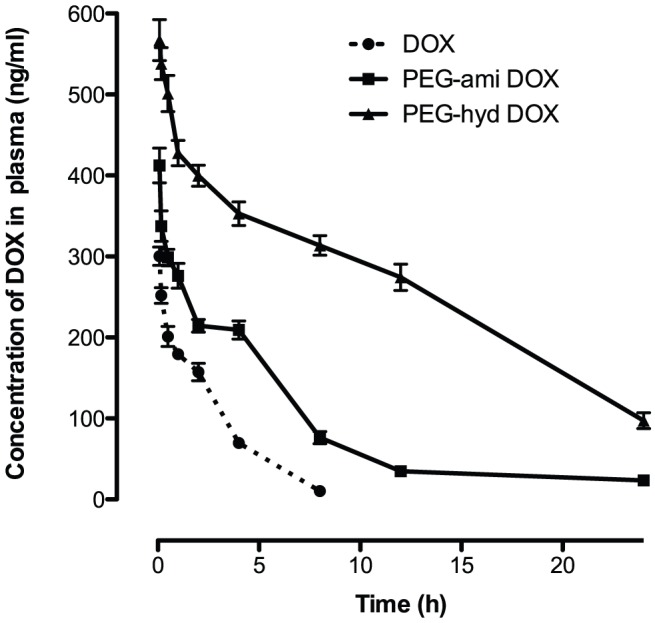
Plasma concentration-time curves of DOX, PEG-ami-DOX and PEG-hyd-DOX after i.v. administration to female tumor-bearing mice at the same 5 mg/kg DOX dose. (n = 5, mean ± SD).

The tumor-targeting efficacy of the DOX conjugates were evaluated by detecting its pharmacokinetic process and bio-distribution by HPLC-MS-MS in blood and different organs of mice bearing MDA-MB-231 cells, respectively. Compared with free DOX (Fig. 6a), drug specific accumulation in tumor tissues was significantly increased following treatment with PEG-hyd-DOX (Fig. 6b), while the localization in other healthy organs, especially in the heart, was reduced obviously (p<0.05 or 0.001) (Fig. 6c). As we know, CHF (chronic heart failure) was the most severe side-effect of DOX, so the lower distribution of DOX in heart by its conjugates will be beneficial in clinical trial, which indicated that the DOX conjugates showed well tumor-targeting activity without severe toxicity. It could also learned that at first 2 h after IV, the difference of DOX accumulation in tumor tissues between free DOX and its conjugates was not significantly (Fig. 6d) however, the accumulation of free DOX distributed to tumor by PEG-hyd-DOX showed peak concentration 8 h after iv; and then decreased to the initial levels after 24 h. On contrary, distribution of DOX formulation in healthy organs gave a decreasing trend accompany by time from 2 to 24 h. These results were consistent to the LC/MS/MS detection, identified PEG-hyd-DOX was a long-circulation profile and the DOX level was still much higher even after 24 h (p<0.001, compared with DOX). The pharmacokinetic process of free dox and two conjuates in plasma were shown in Figure 7. The half-life of PEG-hyd-DOX and PEG-ami-DOX were 7.14 and 9.33 h, respectively. The results were consistent to our previous data and expected this PEG-DOX conjugate to be a potential carrier with tumor-targeting, enhanced antitumor efficacy, long-circulation, and low toxicity profiles.

## Discussion

In the present study, we show this pH-triggered PEG-hyd-DOX conjugate could target DOX to tumor tissues and release free drugs by acidic tumor environment, which would be potent in antitumor drug delivery, and the primary findings regarding above effects are presented as follows: (1) PEG-hyd-DOX have the good anti-tumor effects in *vitro* and *vivo*; (2) PEG-hyd-DOX can targeted tumor cells through pH-triggered effects; (3) PEG-hyd-DOX have the less side effects on normal tissues; (4) PEG-hyd-DOX expressed the characteristic of slow released and long term circulation in *vitro* and *vivo*; (5) Hydrolysis of hydrazone bond in tumor cells could release free DOX to its target sub-cellular region (nucleus) as our designed. Thus, we conclude that of a novel pH-activated polymeric drug delivery system for doxorubicin was established successfully with good antitumor activity in *vitro* and in *vivo*.

During the synthetic route, the functionalized PEG was very vital for the following drug conjugation, so lots of groups have been introduced to the terminal of PEG [22]. In this paper, based on the structure of DOX, we made PEG carboxyl functionalized for next conjugation. Lots of strategies such as anhydride had been applied for carboxylation. However, they would change the basic structure of polymer and made these modified polymers become unstable, which might be hydrolysis before free drug released. So here we chose oxidation in presence of KMnO_4_ to synthesis the functional carboxylation PEG without backbone structure change, the reaction was easy to control and with a high yield of 80%.

pH-triggered drug delivery system has merged as an ideal carrier for anti-tumor agents, related to the acidic extracellular microenvironment in tumor tissues and some acidic organelles like lysosome and endosome. In our study, we synthesized a pH-sensitive DOX conjugate via a chemical linkage by hydrazone, and confirmed its profile by release behavior *in vitro*. From the results we learned that our conjugate was much acid-sensitive at pH 5.0 than 6.8, meant it might be an intracellular pH-triggered carrier. This characteristics attracted great interest in DOX delivery to tumor cells, after vein injection the polymeric conjugates will stay stable during blood circulation at physiological condition, resulted in a low systemic toxicity; once achieved and uptake by tumor cells, there will be a burst release of DOX for the acidic environment of lysosome-endosome, exhibiting a high intracellular concentration of DOX and obviously improved antitumor efficacy. This kind of carriers could be stable until uptake by tumor cells, made few DOX released before arrived at target region, led to low toxicity to normal cells.

DOX is positive for its free amid group with lone pair electrons, once entered the tumor cells, the DOX-conjugates, for its high molecule, would be first distributed in lysosome-endosome and be locked for the electrostatic attraction,if the drugs couldn't escape, they might lose their activity eventually [9]. However, PEG-hyd-DOX could change this embarrassment by their pH-liable hydrazone linkage, when the hydrolysis of hydrazone bond occurred at low pH conditions in lysosome-endosome, lots of proton were acquired accompany with chloride ion flowing into the cells, led to lysosome swelling, cracking and unlocking the drugs, which was called as proton sponge effect [23]. So the pH-triggered profile of PEG-hyd-DOX could let a burst release of DOX when acidic and made free DOX localized to nucleus by proton sponge effect. The fluorescence images convinced our hypothesis by the co-localization of red fluorescence (DOX) with DAPI in nucleus (purple), confirmed PEG-hyd-DOX could escape from acidic organelles and finally arrived at nucleus. However, PEG-ami-DOX didn't show the stimuli sensitive behavior, so it would be locked in cytoplasm (localization of red fluorescence in cytoplasm), which might result in a low therapeutic efficacy. Such image data was not enough to evaluate the qualification level of DOX, and thus the intracellular accumulation of free DOX, PEG-ami-DOX and PEG-hyd-DOX were detected and evaluated using HPLC/MS/MS methods in the following experiments.

Because DOX was an intracellular chemotherapy agent, so the intracellular accumulation of DOX in tumor cells could be seemed as an effective dose in cytotoxocity [24]. We determined the intracellular accumulation by HPLC/MS/MS method, which testified as high sensitive, good accuracy and reproducibility. From the HPLC/MS/MS analysis, as for the pH-stimuli release profile, PEG-hyd-DOX showed a highest accumulation in all the three tumor cells than both PEG-ami-DOX and free drug. PEG-ami-DOX and PEG-hyd-DOX were released slowly which has been demonstrated in the section of drug released experiment, and thus, above two different methods revealed the common phenomenon that these two conjugates can be released slowly in tumor microenvironment as our original designed. Consistent to the *in vitro* release profile, PEG-hyd-DOX remain its structure when extracellular, once in tumor cells, this conjugate would become unstable and release free drugs due to its high sensitivity to low pH in lysosome-endosome. The high intracellular concentration of DOX might be a premise for good toxicity against tumor cells and enhanced anti-tumor activity *in vivo*, indicated our designed conjugate will be more toxic than free DOX. Meanwhile, PEG-ami-DOX performed well than free DOX, explained by the different uptake mode by tumor cells. Polymers were often taken into cells via endocytosis, whereas free DOX, a small molecule drug, would passively diffuse into intracellular regions freely and quickly. It could also be learned that the intracellular DOX accumulation by those high molecule conjugates was increased with incubation time, consistent to their slow-released profile and long-acting effect, which might be guidance in dosage regimen assessment.

Cytotoxicity is a directly index for anti-tumor activity evaluation [25]. Considered the mechanism of action of DOX, a high accumulation of DOX in tumor cells might relate to a well toxicity. As known from the HPLC/MS/MS analysis, both two conjugates showed high intracellular concentration than free DOX, but their toxicity was significant different. PEG-hyd-DOX performed much better in cell growth inhibition than amid conjugate. Even PEG-ami-DOX also gave a higher accumulation of DOX than free drug in tumor cells due to its different uptake mode; it still showed the least toxicity among those formulations. Without pH-stimuli profile, although free DOX released from PEG-ami-DOX, they would be locked in lysosome-endosome (red dots observed in cytoplasm) and couldn't arrive to nucleus to take effect; However, PEG-hyd-DOX took on best toxicity illustrated by the enhanced accumulation of free DOX in tumor cells with help of pH-triggered profile and a following sub-distribution into nucleus by proton sponge effect. The good cytotoxicity of PEG-hyd-DOX further proved the effect of pH-stimuli in tumor therapy. These results indicated than an ideal drug carrier should combine three features: keep stable before targeting site; release free drug completely when necessary; help transporting free drug to targeting region if possible. Fortunately, PEG-hyd-DOX met all these demands, so it hoped to be potent in antitumor therapy.

The results of drug released have been showed that the release of PEG-ami-DOX was much lower than PEG-hyd-DOX in tumor environment, this was mainly due to the different sub-distribution of such conjugates in tumor cells. In addition, the intracellular accumulation of PEG-hyd-DOX and PEG-ami-DOX also expressed different manners in the same condition. This direct evidence explain the reason why PEG-hyd-DOX have the better anti-tumor effect and less side-effect than PEG-ami-DOX, and also agreed with our original designing ideas. And thus, PEG-hyd-DOX was selected as the good candidate for the evaluation of vivo antitumor activity. DOX concentration was detected by HPLC/MS/MS to reflect the release level of PEG-hyd-DOX which was designed to release slowly in tumor, and our results also showed that PEG-hyd-DOX gave a long-circulation profile and retained higher concentration in tumor tissues at 8 hours. And the PEG-hyd-DOX release also expressed the obvious time-dependant manner in vitro experiments. Combine above evidences, such slowly released characteristics will be the base for the long-acting anti-tumor effects and lower side-effects. For another, such slowly released action was related with the intracellular uptake mode of polymer conjugate which often taken into cells via endocytosis, but for free DOX, will passively diffuse into intracellular regions freely and quickly due to its small molecule weight.

Based on our results from *vivo* antitumor activity, there are no significant difference in therapeutic effects, such as tumor volume, of different dose group before 28 days of the first injection, but the difference were clearly among these three dose groups after 28 days of the first injection. In the higher dose groups (10 mg/kg, 15 mg/kg), the tumor volumn were lower than other two groups (p<0.05 or 0.01, Figure S3). And combined the results in *vitro*, both evidence showed the therapeutic effects of PEG-hyd-DOX with time-dependtant manner. In addition, the body weight changes which reflect the toxicity of conjugates was independent of different dose, and demonstrated the steady and less toxicity of PEG-hyd-DOX in *vivo*. Furthermore, the data of mice survival also supported that PEG-hyd-DOX of each dose group can prolong the medium time differently after 28 days of the first injection (data not shown).

Following the *in vitro* results, we investigated the *in vivo* anti-tumor activity of PEG-hyd-DOX evaluated by tumor volume, weight change and survival time. A smaller tumor volume is the confirmed proof of tumor inhibition, so the decrease of tumor size compared with free DOX showed an improved anti-tumor efficacy and superiority than free DOX, consistent to its good toxicity *in vitro*. The EPR effect of polymer conjugate, enhanced intracellular DOX concentration and proton sponge effect due to hydrazone bond were responsible to this result. What's more, PEG-hyd-DOX could prolong mice survival time a lot than free DOX in all their three treatment groups, might result in good prognosis in clinical trial. Weight loss was a common side-effect during chemotherapy led to poor quality of life [26]. PEG-hyd-DOX, however, didn't show this side-effect, proved to be a more safety and low systemic toxicity delivery system. Its well safety made a higher fixed dose for better therapeutic efficacy feasible. The bio-distribution of PEG-hyd-DOX revealed its tumor-targeting activity; PEG-hyd-DOX was passive tumor targeting by EPR effect and environment responsive targeting by its pH-liable release. Many conjugates showed well toxicity *in vitro*, however, their *in vivo* antitumor activity was not good or they didn't perform tumor-targeting profile as expected, which was mainly caused by their instability during blood circulation. With pH-sensitive release profile via hydrazone bond, PEG-hyd-DOX could stay stable till to tumor tissue, made their tumor targeting possible. The tumor targeting activity meant our designed conjugate could be specific target to tumor tissue and made a high effective dose, might lead to good therapeutic efficacy and low toxicity. PEG-hyd-DOX was an intracellular pH-triggered conjugate; its antitumor efficacy depended on high intracellular concentration of free drugs in target region. As described *in vitro* results before, once uptake by tumor cell, free drug released from the conjugate immediately and escaped from cytoplasm to nucleus to take effect. So the pH-stimuli profile was crucial for either tumor-targeting or antitumor efficacy *in vivo*. All these *in vivo* data indicated PEG-hyd-DOX would be a potential drug delivery system for chemotherapy agents.

The preparation and evaluation of a DOX conjugate, PEG-hyd-DOX, via a hydrazone linkage is described. This DOX conjugate showed pH-triggered release, higher cellular accumulation of DOX, and a higher cytotoxicity *in vitro*. This pH-sensitive conjugate could first distribute to cytoplasm and then made a burst release and unlock DOX from the cytoplasm to nucleus due to its pH-liable profile. Consistent with these results, PEG-hyd-DOX was more effective in tumor therapy than was free DOX and demonstrated good tumor targeting *in vivo*. These results indicate that this DOX conjugate shows good antitumor activity with less systemic side effects.

## Materials and Methods

### Ethics Statement

All procedures had the approval of the Animal Ethics Committee of the Fourth Military Medical University.

### Materials

PEG (MW 6000), doxorubicin hydrochloride (DOX.HCl) and 3-(4,5-dimethylthiazol-2-yl)-2,5-diphenyl tetrazolium bromide (MTT) were obtained from Sigma-Aldrich (St. Louis, MO). 1-ethyl-(3-dimethylaminopropyl)-carbodiimide hydrochloride (EDCI) was obtained from Fluka (Sigma-Aldrich, St. Louis, MO); *t*-butyl carbazate was purchased from Alfa Aesar (Ward Hill, MA). All other chemicals were analytical grade without further purification. The cell lines HepG-2 (human hepatocellular liver carcinoma), MCF-7 and MDA-MB-231 (breast carcinoma) were obtained from Institute of Biochemistry and Cell Biology, Chinese Academy of Science. The severe combined immune deficient mice (SCID) were obtained from experimental animal center of the Fourth Military Medicinal University.

### Synthesis of two PEG- DOX conjugates

The synthesis of PEG-hyd-DOX was achieved in three steps (Fig. 1). Briefly, polyethylene glycol was oxidized to polyethylene glycol dioic acid to obtain terminal carboxylic acid functional groups. Next, *tert*-butyl carbazate (BOC-hydrazide) was conjugated to the modified polymer in the presence of EDCI and TEA (triethylamine) yielding PEG-Hyd-BOC (PEG dihydrazide with BOC protected). The BOC protective group was removed with HCl in ethyl acetate to obtain PEG-Hyd (PEG dihydrazide). DOX was then conjugated to PEG-Hyd, via a hydrazone bond, at the C13 carbonyl group of DOX by reaction with trifluoroacetic acid in methanol for 12 h.

PEG-ami-DOX was synthesized via amid bond for control. Briefly, PEG was firstly functionalized as described above, and then DOX was conjugated to the modified polymer (polyethylene glycol dioic acid) via amid bond at its free amino group with the presence of TEA and catalyzed by EDCI in DCM (Dichloromethane) for overnight.

All reactions was performed under nitrogen and monitored by thin-layer chromatography. The resulting products were purified using a Sephadex G-50 column. After drying, the conjugate products, red powder, were characterized by NMR spectroscopy and HPLC.

### Characterization of the conjugates

The two synthesized conjugates were characterized by ^1^H NMR spectroscopy (INOVA-400 MHz, Varian USA), using CDCl_3_ as solvent. The DOX content were determined by UV spectroscopy at 480 nm immediately after hydrolysis of the conjugate in 1 M HCl at 85°C for 15 min, followed by neutralization. The purity of the conjugate was evaluated by RP-HPLC and the chromatographic separation was performed on a Waters symmetry C18 column (250×4.6 mm, 50 µm; Waters Corporation, Milford, MA, USA) with an isocratic mobile phase of acetonitrile/0.1% aqueous acetic acid (70/30, V/V) at a flow rate of 1 ml/min on Waters (Waters, MA, USA) 2695 HPLC system.

### In vitro release of DOX from PEG-hyd-DOX conjugate

The release of DOX from the PEG-hyd-DOX or PEG-ami-DOX *in vitro* was performed at pH 5.0, 6.8 and 7.4 to investigate the drug release and acid sensitivity characteristics. 20 µg/mL PEG-hyd-DOX and PEG-ami-DOX conjugates (measured as DOX equivalent) were dissolved in acetate buffer saline (pH 5.0), phosphate buffer (pH 6.8 and 7.4) and incubated with gentle shaking in water bath at 37°C. At predetermined intervals (0.5, 1, 2, 4, 6, 8, 12 and 24 h), 20 µL samples were removed and replaced with an equal volume of buffer. The amount of released DOX was determined by HPLC method as described above.

### Cell culture conditions

MCF-7, MDA-MB-231 and HepG2 cells were grown in DMEM (high glucose) supplemented with 10% fetal bovine serum, 1 mM sodium pyruvate, 5 µg/ml insulin (Sigma, St. Louis, MO), 100unit/ml penicillin, 100 µg/ml streptomycin and 25 µg/ml amphotericin B (Invitrogen, Carlsbad, CA). Cultures were maintained in a humidified atmosphere of 5% CO_2_ at 37°C. The cells were subcultured at 80% confluence in 75-cm^2^ tissue culture flasks. For all experiments, 96-well or 24-well plates were inoculated with aliquots of cells removed from the flasks by brief treatment with 0.25% (v/v) trypsin (Invitrogen, Carlsbad, CA) and allowed to grow for 24 h for later drug treatment.

### Cellular sub-distribution of PEG-hyd-DOX conjugate

Cellular sub-distribution of free DOX and its PEG conjugates were studied using a fluorescence microscope (Nikon DS-5M-ui80i Japan) to observe the influence of designed delivery system on intracellular distribution of DOX. Cells were seeded into 24-well plates at a cell density of 1×10^5^ cells/ml. After 24 h, 20 µM free DOX or DOX conjugates (measured as free DOX equivalent, drugs were all dissolved in serum free medium with DMSO less than 0.1% when necessary) were added and incubated for 30 min at 37°C. After drug treatment, the medium was discarded; cells were rinsed three times with PBS, and then treated by 1 µg/ml DAPI for nuclei staining for 10 min, rinsed with PBS for three times and finally fixed with 4% paraformaldehyde for 10 min and observed using the intrinsic fluorescence of DOX by fluorescence microscope.

### LC/MS/MS assay for intracellular DOX accumulation

Intracellular DOX accumulation was quantitatively analyzed by LC/MS/MS. MCF-7, MDA-MB-231 and HepG2 cells were seeded in 24-well plates as described above. Following the treatment with 20 µM free DOX or DOX conjugates (measured as free DOX equivalent, drugs were treated as described above) for 2, 4 and 8 h respectively, the medium was discarded, cells were washed three times with PBS and detached from the plates using trypsin, then centrifuged for 5 min at 1000× *g* to discard the supernatant, and resuspended in purity water. Cells were finally lysed by subjecting them to three freeze-thaw cycles in liquid nitrogen. Cell lysate samples (0.4 ml) were extracted with 1 ml ethyl acetate (0.1 ml of 50 ng/ml resveratrol was included as an internal standard) by vortexing vigorously for 1 min and then centrifuging at 3000× *g* at room temperature for 10 min. Supernatants were evaporated to dryness under nitrogen, reconstituted in 100 µl of methanol and analyzed by LC/MS/MS. Meanwhile, another cell lysate (0.4 ml) were collected to determine the amount of total protein in cells using coomassie brilliant blue. Cellular accumulation of PEG-hyd-DOX was normalized with respect to total protein content in tumor cells [16].

### *In vitro* cytotoxicity

The tetrazolium dye (MTT) assay was performed to determine the cytotoxicity of free DOX and its conjugates in MCF-7, MDA-MB-231 and HepG2 cells based on a previously described method with minor modifications [17]. In brief, 200 mL aliquots of each type of cell suspension (1×10^4^ cells), harvested during a logarithmic growth phase, were pipeted into 96-well round-bottomed plates (Corning Costar, Corning, NY). Each plate was incubated with various concentrations of free DOX or its conjugates for 72 h at 37°C in a humidified atmosphere of 5% CO_2_. Control cells received an equivalent volume of fresh medium. The MTT assay was performed, and the percentage of viable cells was determined. The absorbance of alive cell in each well was measured at 570 nm using a CODA Automated EIA Analyzer (Bio-Rad Laboratories, Hercules, CA).

Based on these measurements, the half-maximal inhibitory concentrations (IC_50_), *i.e.*, the amount of DOX needed to inhibit cell growth by 50%, were calculated for free DOX and its conjugates. A decrease in the IC_50_ value indicates an increase in drug toxicity. The cell inhibition rate was calculated from the absorbance reading of the wells using the following formula:
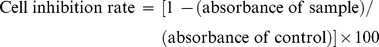



### Inhibition of tumor growth *in vivo*

PEG-hyd-DOX conjugate was used to treat SCID mice bearing MDA-MB-231 breast tumor cells. Female tumor-bearing mice were chosen and divided into different groups at random. Mice were inoculated subcutaneously with 1×10^6^ cells in a volume of 0.2 ml serum-free medium. When the tumor was large enough to be palpable (tumor volume = 50 mm^2^), the mice were treated once with DOX or its conjugate at a fixed dosage by tail vein injection and then were treated a second time 7 days later. The mice were observed every 3 days to monitor weight change as a sign of drug toxicity. The therapeutic efficacy was evaluated by tumor size and survival time. Survival time was assessed by a survival curve, and tumor size was measured every 3 days and calculated with the formula below:
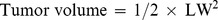
where L and W are the length and width of the tumor, respectively.

### Pharmacokinetic and bio-distribution of PEG-hyd-DOX conjugate

DOX and its conjugate were administered to female tumor-bearing mice by tail vein injection to examine their plasma concentration and tissues distribution. After injection the mice were sacrificed, and the blood (at time of 0.08, 0.17, 0.5, 1, 2, 4, 8, 12, 24 h) and organs (tumor, heart, liver, spleen, lung, and kidney at fixed time intervals of 2, 8 and 24 h) were collected. Tissues were weighed and mixed with acetonitrile (with 0.1 ml of 50 ng/ml resveratrol added as an internal standard) and then homogenized. The sample of blood and homogenate from the different tissues were centrifuged at 3000× *g* for 10 min, and the supernatants were collected and analyzed for DOX contents by HPLC/MS/MS.

### Statistical analysis

All experiments were performed in quadruplicate. The results are expressed as the mean ± SD from four to six independent measurements. Statistical analysis was performed as a one-way analysis of variance (ANOVA), and comparisons among groups were performed using an independent sample t-test.

## Supporting Information

Figure S1 Characterization of synthesized conjugates. ^1^H NMR spectra of (a) PEG-hyd-DOX in CDCl_3_ and (b) PEG-ami-DOX CDCl_3_; HPLC chromatogram for purity of (c) PEG-hyd-DOX and (d) PEG-ami-DOX.
(EPS)

Figure S2 Particle size distribution of synthesized conjugates. The mean particle size which was measured by Zetasizer Nano instrument were 140 and 160 nm for PEG-ami-DOX (a) and PEG-hyd-DOX (b), respectively.
(EPS)

Figure S3 Evaluation of in vivo anti-tumor activity of free DOX and PEG-hyd-DOX (5,10,15 mg/kg DOX. eq) on nude mice after s.c.human breast MDA-MB-231 cells (between 14 to 28 days).
(EPS)
